# A Self-Assembled Metabolic Regulator Reprograms Macrophages to Combat Cytokine Storm and Boost Sepsis Immunotherapy

**DOI:** 10.34133/research.0663

**Published:** 2025-04-01

**Authors:** Junyan Zhuang, Yongrui Hai, Xintong Lu, Borui Sun, Renming Fan, Bingjie Zhang, Wenhui Wang, Bingxue Han, Li Luo, Le Yang, Chun Zhang, Minggao Zhao, Gaofei Wei

**Affiliations:** ^1^ Institute of Medical Research, Northwestern Polytechnical University, Xi’an 710072, China.; ^2^ Research & Development Institute of Northwestern Polytechnical University in Shenzhen, Shenzhen 518057, China.; ^3^ Department of Anesthesiology, The First Affiliated Hospital of Xi’an Jiaotong University, Xi’an 710061, China.; ^4^ Department of Pharmacy, Tangdu Hospital, Air Force Military Medical University, Xi’an 710038, Shaanxi, China.; ^5^ Department of Surgical Intensive Care Unit, The First Affiliated Hospital of Xi’an Jiaotong University, Xi’an 710061, China.

## Abstract

Sepsis, a life-threatening inflammatory disorder characterized by multiorgan failure, arises from a dysregulated immune response to infection. Modulating macrophage polarization has emerged as a promising strategy to control sepsis-associated inflammation. The endogenous metabolite itaconate has shown anti-inflammatory potential by suppressing the stimulator of interferon genes (STING) pathway, but its efficacy is inhibited by hyperactive glycolysis, which sustains macrophage overactivation. Here, we revealed a critical crosstalk between the itaconate–STING axis and glycolysis in macrophage-mediated inflammation. Building on this interplay, we developed a novel nanoparticle LDO (lonidamine disulfide 4-octyl-itaconate), a self-assembled metabolic regulator integrating an itaconate derivative with the glycolysis inhibitor Lonidamine. By concurrently targeting glycolysis and STING pathways, LDO reprograms macrophages to restore balanced polarization. In sepsis models, LDO effectively attenuates CCL2-driven cytokine storms, alleviates acute lung injury, and significantly enhances survival via metabolic reprogramming. This study offers a cytokine-regulatory strategy rooted in immunometabolism, providing a foundation for the translational development of immune metabolite-based sepsis therapies.

## Introduction

Sepsis is a fatal syndrome characterized by life-threatening organ dysfunction originating from a chaotic and unchecked innate immune response to infection [1,2]. Notably, macrophages are the master regulators of inflammation and immune homeostasis [3,4]. However, macrophages become hyperactivated in septic conditions, flooding the system with pro-inflammatory cytokines that fuel immune dysregulation and precipitate cascading organ failure [5–7]. In particular, sepsis-induced acute lung injury has become a focus of contemporary research [8,9]. Therefore, the immunomodulatory capacity and polarization of macrophages are intricately associated with the onset and progression of sepsis.

Macrophage metabolism is crucial in this polarization because the shift from oxidative phosphorylation to glycolysis powers the inflammatory M1 phenotype during sepsis [10,11]. This metabolic shift enables macrophages to meet the bioenergetic and biosynthetic demands of the immune surge [12,13]. Meanwhile, multidimensional regulatory pathways of macrophage polarization and function, including immunometabolism, epigenetics, and immune checkpoints, have been clearly elucidated [14–16]. Consequently, targeting glycolysis represents an innovative therapeutic strategy for manipulating macrophage function and potentially reprogramming the inflammatory response to mitigate sepsis-induced damage.

Itaconate, a metabolite deeply embedded in the macrophage metabolism [17], has garnered significant attention due to its regulatory role [18,19]. Studies have demonstrated that itaconate suppresses the NOD-like receptor thermal protein domain associated protein 3 (NLRP3) inflammasome activation [20,21]. Moreover, its derivative 4-octyl itaconate (4-OI) shows considerable efficacy in attenuating sepsis by blocking the stimulator of interferon genes (STING) innate immunity pathway [22,23]. However, hyperactive glycolysis in macrophages can reduce the anti-inflammatory effects of itaconate during sepsis, potentially restoring a pro-inflammatory profile [24]. Thus, the precise modulation of glycolysis could elucidate the full therapeutic potential of itaconate.

**Fig. 1.**
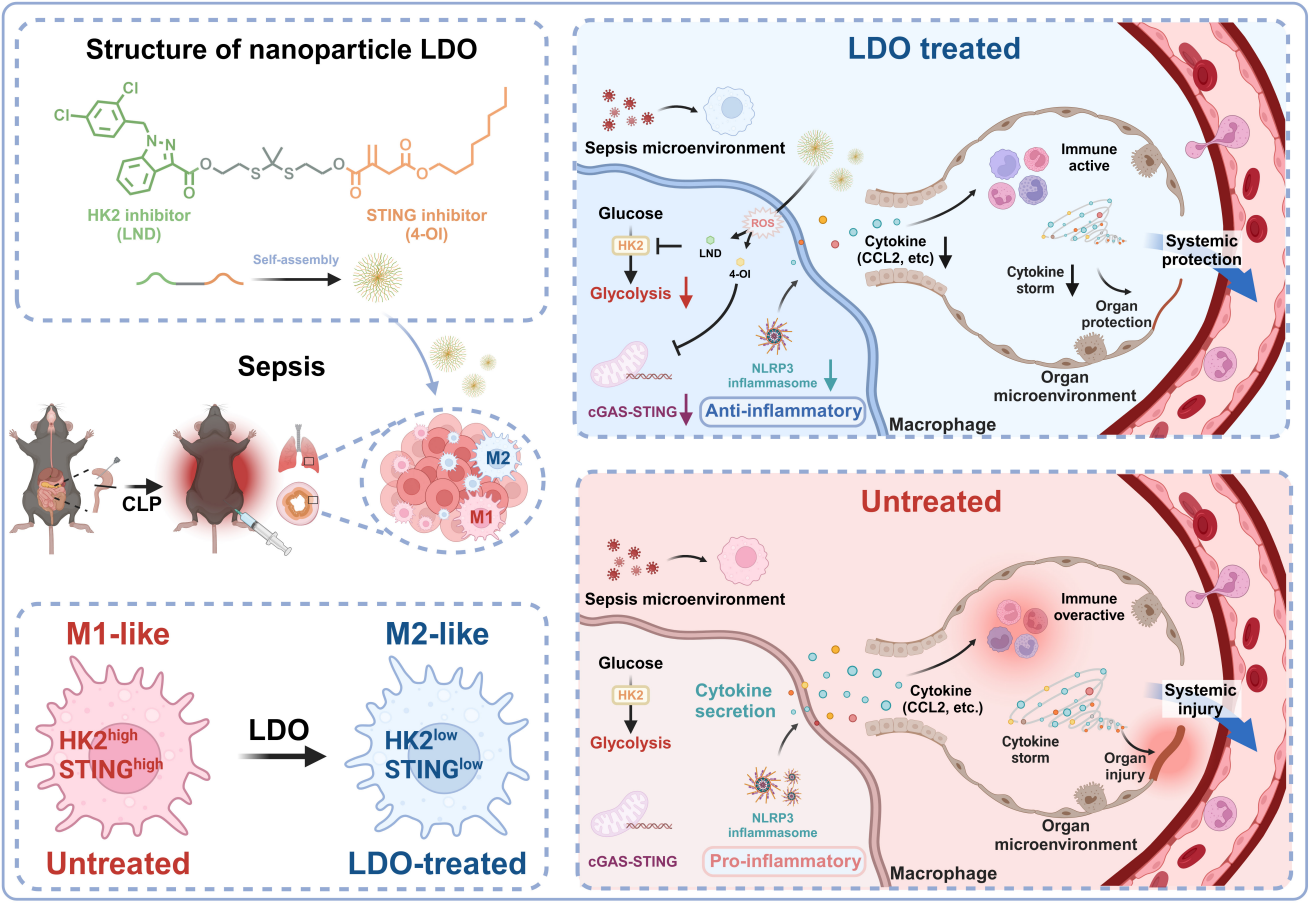
The structure and mechanism of nanoparticle LDO. The schematic illustration of LDO recalibrates macrophage polarization from M1 to M2, inhibiting CCL2-driven cytokine storm, ultimately alleviating sepsis.

In this study, we developed an advanced nanoparticle LDO, which integrates the glycolysis inhibitor Lonidamine (LND) with the STING inhibitor 4-OI [25,26], ultimately enhancing the function of itaconate (Fig. 1). Mechanistically, LDO exhibits a remarkable capacity to recalibrate macrophage polarization, shifting the balance from M1-driven inflammation to M2-mediated repair. Furthermore, LDO blockade effectively diminishes the production of chemokine CCL2, a critical orchestrator of the cytokine storm underlining septic pathology [27]. Overall, our findings reveal the profound therapeutic potential of LDO, positioning it as a groundbreaking agent capable of reinforcing macrophage-driven inflammation, safeguarding organ integrity, and reshaping the treatment paradigm for sepsis.

## Results

### Therapeutic potential of HK2 and STING-mediated macrophages metabolic reprogramming in sepsis

**Fig. 2.**
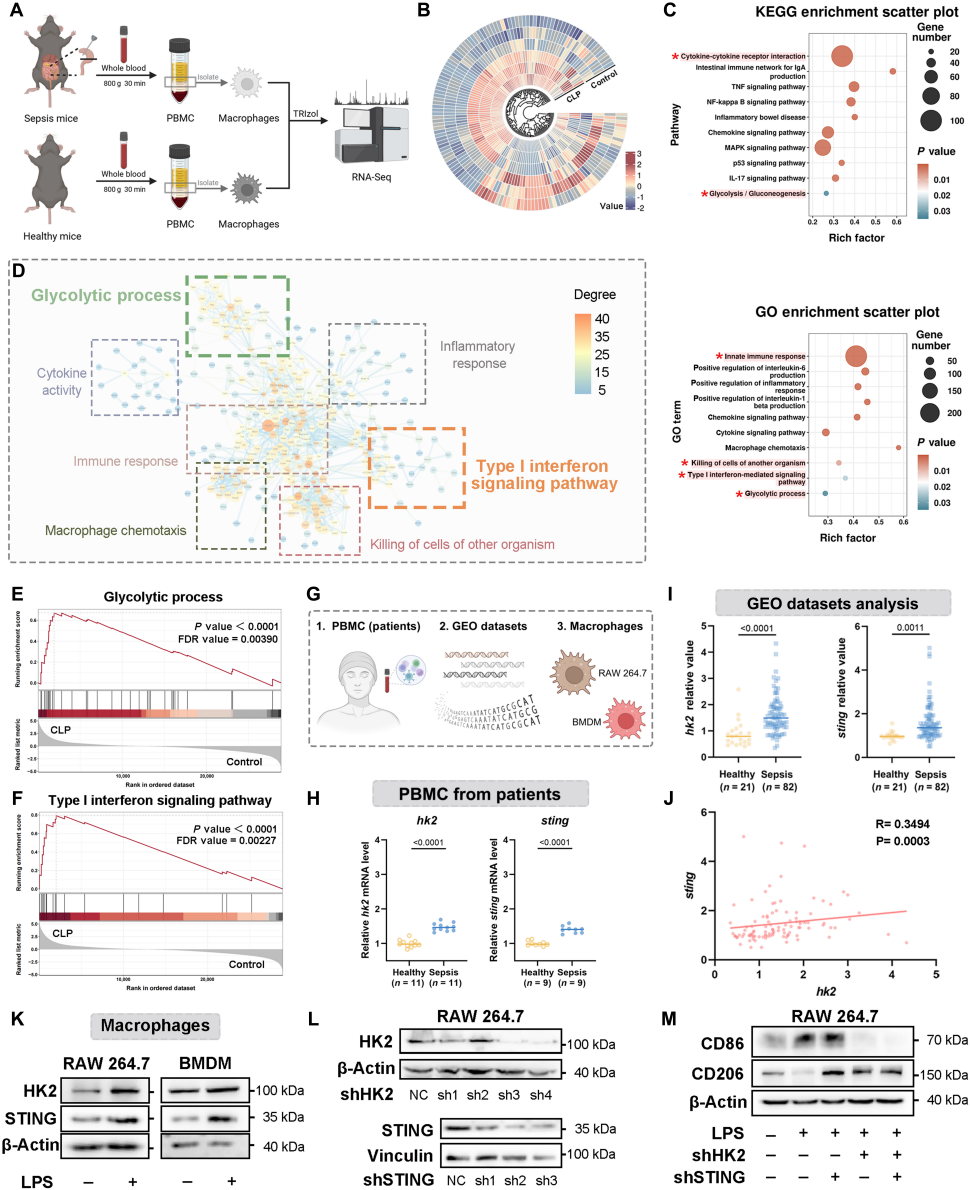
Therapeutic potential of HK2 and STING-mediated macrophages metabolic reprogramming in sepsis. (A) Schematic overview of the experimental design of RNA-seq. (B) Heatmap of differentially expressed genes in sepsis or healthy mice. (C) KEGG and GO enrichment analysis of differentially expressed genes in sepsis or healthy mice. (D) A network diagram depicts the relationships between differentially expressed pathways and their associated genes. (E and F) GSEA of genes in glycolytic process (E) and type I interferon signaling pathway (F). (G) Experimental establishment for investigating HK2 and STING in sepsis or healthy condition. (H) *hk2* and *sting* expression in PBMCs from patients. (I and J) Bioinformatic analyses of *hk2* and *sting* expression (I) and their correlation (J) in GEO datasets (GSE26378). (K) Western blot for HK2 and STING in RAW264.7 cells and BMDMs with or without LPS treatment. (L) Western blot for HK2 and STING in RAW264.7 cells after shRNA-mediated knockdown. (M) Western blot for CD86 and CD206 in *hk2*, *sting*, or co-knockdown RAW264.7 cells with or without LPS treatment.

Initially, to investigate the metabolic alterations of macrophages during sepsis, we employed cecal ligation and puncture (CLP) mouse sepsis model to explore metabolic alterations caused by macrophages [28,29]. Notably, fresh blood was harvested from the mice 24 h postoperatively, and macrophages from peripheral blood mononuclear cells (PBMCs) were isolated for RNA sequencing (RNA-seq) analysis (Fig. 2A). Transcriptomic analysis revealed 4,567 significantly differentially expressed genes in the sepsis group (Fig. 2B and Fig. S1A), with a high intragroup correlation (Fig. S1B). Kyoto Encyclopedia of Genes and Genomes (KEGG) and Gene Ontology (GO) enrichment analyses identified significant enrichment in pathways associated with cytokine–cytokine receptor interaction, glycolysis, type I interferon signaling, and organ injury (Fig. 2C). Meanwhile, gene-level correlation analyses based on metabolic characteristics were conducted on these enriched pathways (Fig. 2D), indicating that glycolysis and STING-dependent type I interferon release in macrophages could be highly correlated with the severity of sepsis.

Furthermore, we conducted a series of focused investigations on glycolysis and STING-dependent type I interferon signaling pathways. Gene Set Enrichment Analysis (GSEA) demonstrated a significant up-regulation of these pathways in macrophages (Fig. 2E and F). Similarly, the expression levels of critical genes associated with glycolysis were analyzed (Fig. S1C). Hexokinase 2 (HK2), an essential rate-limiting enzyme in glycolysis [30], was significantly up-regulated in PBMCs, underscoring its pivotal role in macrophage metabolic reprogramming during sepsis.

To assess the clinical therapeutic potential of HK2 and STING, comprehensive experiments were carried out from clinical patient samples to cellular models, including mouse monocyte macrophage cell line RAW264.7 and mouse bone marrow-derived macrophages (BMDMs) (Fig. 2G). In summary, similar results were confirmed in patient PBMCs (Fig. 2H), bioinformatic analyses (Fig. 2I), and macrophages (Fig. 2K). Likewise, a positive correlation between *hk2* and *sting* was identified in septic mice based on Gene Expression Omnibus (GEO) datasets (Fig. 2J).

Based on these observations, RAW264.7 cells were generated with shRNA-mediated knockdown of *hk2*, *sting*, or both in order to investigate the role of *hk2* and *sting* in macrophages (Fig. 2L). The co-knockdown of *hk2* and *sting* significantly reduced CD86, a pro-inflammatory marker, while enhancing the expression of the anti-inflammatory marker CD206 (Fig. 2M). Moreover, inflammatory cytokine interleukin-1β (IL-1β) and IL-6 secretion were significantly reduced (Fig. S1D). These findings suggest that inhibiting HK2 and STING can reverse macrophage polarization toward a pro-inflammatory phenotype, thereby mitigating the excessive inflammatory response characteristic of early sepsis.

### LDO’s self-assembling potential and superior efficacy in alleviating sepsis

**Fig. 3.**
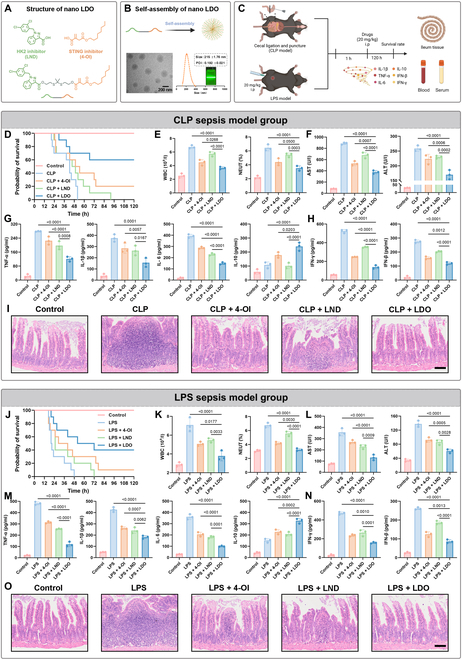
LDO’s self-assembling potential and superior efficacy in alleviating sepsis. (A) Chemical structure and composition of LDO. (B) The nanoparticles were characterized based on their morphology and size distribution; scale bars: 200 nm; PDI, polydispersity index. (C) Experimental design: sepsis or healthy C57BL/6 mice were treated with PBS, 4-OI, LND, and LDO. (D to F and J to L) Survival rate (*n* = 10), blood routine (*n* = 3), and liver function examinations (*n* = 3) were measured after treatment with PBS, 4-OI, LND, and LDO. (G and H, and M and N) The concentrations of cytokines in serum samples, including TNF-α, IL-1β, IL-6, IL-10, IFN-γ, and IFN-β (*n* = 3), were quantified using ELISA. (I and O) Representative H&E-stained sections of ileum tissue. Morphology was examined using light microscopy. Scale bar: 120 μm.

Considering that HK2 and STING expressing macrophages are abundant in sepsis and are associated with drastic inflammation, we developed a novel immunomodulating agent referred to as LDO, which links glycolysis inhibitor LND with STING inhibitor 4-OI to achieve the dual inhibition of HK2 and STING (Fig. 3A). Scheme S1 shows the route of synthesis. The structures of the compounds were confirmed through a rigorous structural analysis using ^1^H-NMR and ^13^C-NMR.

To demonstrate the self-assembly characteristics of the LDO, we conducted Tyndall effects and dynamic light scattering (DLS) experiments. As expected, LDO formed spherical nanoparticles with a diameter of approximately 200 nm (Fig. 3B). Additionally, reactive oxygen species (ROS) reactivity was evaluated using high-performance liquid chromatography (HPLC). Our results evaluated that upon exposure to 10 mM hydrogen peroxide, the dithioketal bond within the nanoparticle significantly degraded, resulting in complete cleavage after 4 h (Fig. S2A). Furthermore, HPLC and DLS analyses demonstrated the stability of the compound in phosphate-buffered saline (Fig. S2B and C).

Next, we analyzed the therapeutic effects of LDO in vivo during CLP-induced and lipopolysaccharide (LPS)-induced sepsis (Fig. 3C). Notably, LND exhibited negligible therapeutic effects in septic cases, whereas 4-OI slightly alleviated symptoms, such as sluggish movement and labored breathing, modestly extending the survival period of mice (Fig. 3D and J). In contrast, the therapeutic effect of LDO on sepsis was most significant.

Simultaneously, routine blood examinations and liver function tests were conducted to evaluate inflammatory damage at the systemic level [31]. Routine blood examinations showed that white blood cells and neutrophil levels significantly improved after LDO treatment in the CLP (Fig. 3E) and LPS model (Fig. 3K), indicating the ability of LDO to alleviate immune dysfunction caused by the excessive activation of the immune response during sepsis. Furthermore, biochemical indicators of liver function revealed that LDO significantly down-regulated alanine transaminase and aspartate transaminase levels in mice with sepsis in the CLP (Fig. 3F) and LPS model (Fig. 3L), indicating a protective effect on liver function.

Moreover, to further assess cytokine storm formation in mice with sepsis, enzyme-linked immunosorbent assay (ELISA) was used to measure the levels of serum pro-inflammatory and anti-inflammatory cytokines. Notably, in the CLP (Fig. 3G) and LPS model (Fig. 3M), LDO significantly inhibited the production of inflammatory cytokines such as tumor necrosis factor-alpha (TNF-α), IL-1β, and IL-6, whereas LDO treatment led to a notable increase in IL-10 levels, indicating the strong anti-inflammatory potential of LDO. Similarly, the type I interferon content was also significantly reduced in the CLP (Fig. 3H) and LPS model (Fig. 3N). Finally, we evaluated the impact of LDO on sepsis-associated organ injury by hematoxylin and eosin (H&E) staining. The results indicated that, in both the CLP (Fig. 3I) and LPS models (Fig. 3O), the LDO group displayed thicker and more robust villi, along with a greater density of lamina propria lymphocytes in the ileal tissues compared to the other groups. However, mice with sepsis exhibit severe intestinal injury and numerous inflammatory cells at the injury site. We also evaluated the in vivo safety of all drugs using H&E staining and routine blood examinations (Fig. S2D and E). Meanwhile, LDO treatment did not significantly alter HK2 expression in lung tissues (Fig. S2F), indicating that LDO does not affect normal tissues.

These results demonstrate that LDO can effectively alleviate sepsis, reduce the early cytokine storm triggered by excessive activation of inflammatory factors, and ultimately improve survival rates in mice with sepsis while ameliorating intestinal injury.

### LDO alleviates sepsis-induced lung injury

**Fig. 4.**
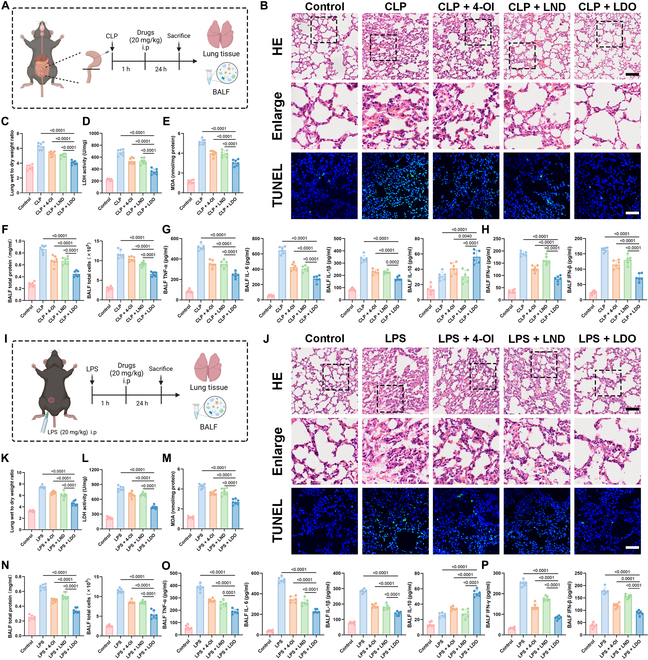
LDO alleviates sepsis-induced lung injury. (A and I) Schematic overview of the experimental design of CLP- or LPS-induced lung injury. (B and J) Representative images of H&E staining of lung tissue. Scale bar: 80 μm. Representative images of TUNEL staining of lung tissue. Scale bar: 65 μm. Morphology was examined using light microscopy. (C to E and K to M) Lung wet/dry ratio (C and K), LDH activity (D and L), and MDA level (E and M) of lung tissues were measured after treatment with PBS, 4-OI, LND, and LDO (*n* = 7). (F and N) The total cell and protein content in the BALF were measured (*n* = 7). (G and H, and O and P) ELISA was used to measure the levels of cytokines (TNF-α, IL-1β, IL-6, IL-10, IFN-γ, and IFN-β) in BALF. (*n* = 6).

The progression of macrophage inflammation was highly correlated with organ injury [32]. Therefore, we chose the lungs, which are among the most vulnerable organs during sepsis [33], as the focus of this study in CLP (Fig. 4A) and LPS mouse septic models (Fig. 4I). Furthermore, to visualize the extent of lung injury, H&E staining and TdT-mediated dUTP-nick end labeling (TUNEL) staining were performed in the CLP (Fig. 4B) and LPS model (Fig. 4J). Compared with the other groups, the LDO-treated group exhibited significantly reduced pathological injury, characterized by regular lung tissues morphology and clear intact alveolar structures.

Furthermore, we evaluated the lung tissues’ wet/dry ratio, lactate dehydrogenase (LDH) activity, and malondialdehyde (MDA) content to assess pulmonary edema [34]. After LDO treatment, the lung tissue wet/dry ratio and LDH was significantly reduced, indicating that LDO effectively inhibits pulmonary edema in the CLP (Fig. 4C and D) and LPS model (Fig. 4K and L). Additionally, LDO significantly reduced MDA levels in the CLP (Fig. 4E) and LPS model (Fig. 4M), suggesting that LDO may alleviate sepsis-induced lung injury by modulating oxidative stress.

Additionally, to gain deeper insight into inflammatory infiltration in the lung tissues, bronchoalveolar lavage fluid (BALF) was isolated to assess the extent of inflammatory infiltration [35]. The total cell and protein contents in the BALF of mice with sepsis were significantly increased in the CLP (Fig. 4F) and LPS model (Fig. 4N), indicating inflammatory cell infiltration and cytokine storm development; however, LDO treatment significantly reduced both. The changes in crucial pro-inflammatory cytokines (TNF-α, IL-1β, and IL-6) and the anti-inflammatory cytokine IL-10 were similar to those observed in the serum from the CLP (Fig. 4G) and LPS model (Fig. 4O). Similarly, the type I interferon levels were significantly reduced in the CLP (Fig. 4H) and LPS model (Fig. 4P). Therefore, our results have demonstrated that LDO attenuates the excessively activated cytokine storm in lung tissues, effectively alleviates sepsis-related lung injury, and reduces the infiltration of inflammatory cells.

### LDO inhibits HK2-mediated glycolysis and cGAS-STING pathway

Following the identification of the crucial in vivo therapeutic effects of LDO, we investigated their effects on macrophages using the RAW264.7 and BMDMs. Firstly, to determine the effective concentration of LDO required to inhibit macrophage inflammatory phenotypes at the cellular level, we assessed the expression levels of the M1 marker CD86 and M2 marker CD206 using flow cytometry and Western blotting (WB) (Fig. S3A and B). At a concentration of 50 μM, LDO significantly down-regulated CD86 expression and up-regulated CD206 expression. These findings suggest that LDO at a concentration of 50 μM can induce macrophage polarization from M1 to M2, thereby reducing macrophage inflammation.

**Fig. 5.**
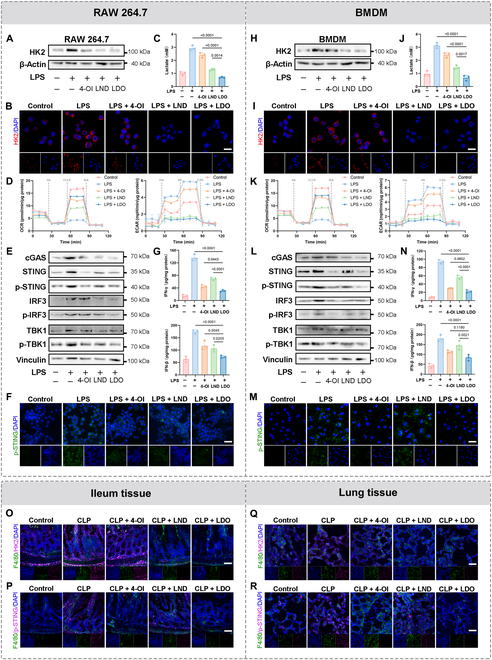
LDO inhibit HK2-mediated glycolysis and cGAS-STING pathway. (A and H) Western blot for HK2 was performed, after treatment of 4-OI, LND, or LDO in RAW264.7 cells and BMDMs. (B and I) Immunofluorescence of HK2 in RAW264.7 cells and BMDMs. Scale bars: 20 μm. (C and J) Lactate level in RAW264.7 cells and BMDMs after treatment of 4-OI, LND, or LDO. (D and K) Real-time OCR and ECAR of RAW264.7 cells and BMDMs. (E and L) Western blot results for STING signaling cascades. (F and M) Immunofluorescence of p-STING in RAW264.7 cells and BMDMs. Scale bars: 30 μm. (G and N) ELISA was used to measure type I interferons (IFN-γ and IFN-β) in medium. (O to R) Immunofluorescence of HK2 and p-STING in ileum and lung tissue. Ileum scale bars: 50 μm. Lung scale bars: 25 μm.

As evidenced through WB and immunofluorescence (IF), HK2 inhibition was verified at the protein level in RAW264.7 (Fig. 5A and B) and BMDMs (Fig. 5H and I). The levels of the glycolytic metabolite lactate significantly reduced in RAW264.7 (Fig. 5C) and BMDMs (Fig. 5J). We also assessed glycolysis using the oxygen consumption rates (OCRs) and extracellular acidification rates (ECARs) to demonstrate that LDO inhibits glycolysis in RAW264.7 (Fig. 5D) and BMDMs (Fig. 5K). In summary, consistent with the other treatments, LDO maintained HK2 expression levels equal to or lower than those of the control group.

Furthermore, an investigation of the STING-related mechanism revealed that LDO down-regulated the phosphorylation levels of STING signaling pathway components [36], including cyclic GMP-AMP synthase (cGAS), TANK-binding kinase 1 (TBK1), and interferon regulatory factor 3 (IRF3) in RAW264.7 (Fig. 5E) and BMDMs (Fig. 5L). IF staining comprehensively assessed phosphorylated STING (p-STING) activation (Fig. 5F and M). Meanwhile, the levels of pro-inflammatory factors, anti-inflammatory factors, and type I interferons in the medium showed a similar trend after LDO treatment (Fig. 5G and N and Fig. S3C).

Additionally, the excellent in vivo capability of LDO for dual inhibition of HK2 and STING was revealed in the ileum and lung tissue slices (Fig. 5O to R). Meanwhile, the down-regulation of HK2 and STING was observed in PBMCs (Fig. S3D). Overall, these results indicate that LDO modulates the production of macrophage-related inflammatory factors by inhibiting HK2 and STING, both in vivo and in vitro.

### LDO modulates cytokine signaling and promotes M2 macrophage polarization to alleviate septic inflammation

**Fig. 6.**
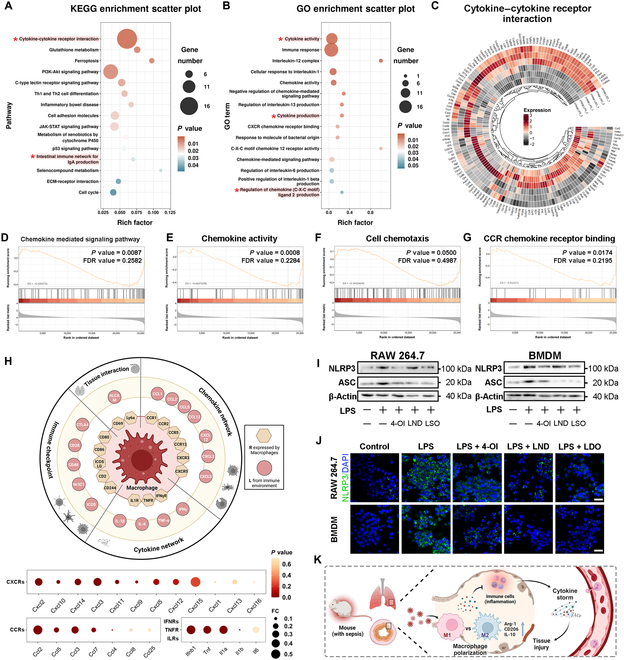
LDO modulates cytokine signaling and promotes M2 macrophage polarization to alleviate septic inflammation. (A and B) KEGG (A) and GO (B) enrichment analysis of differentially expressed genes in BMDMs after LPS or LPS + LDO treatment. (C) Heatmap of main genes in the cytokine–cytokine receptor interaction pathway (*n* = 3). (D to G) GSEA of genes in chemokine-mediated signaling pathway (D), chemokine activity (E), cell chemotaxis (F), and CCR chemokine receptor binding (G) pathway. (H) R–L network in macrophages during sepsis. (I) Western blot for NLRP3 and ASC in RAW264.7 cells and BMDMs. (J) Immunofluorescence of NLRP3 in RAW264.7 cell and BMDMs. Scale bars: 30 μm. (K) Scheme of LDO’s therapeutic mechanism.

Furthermore, to gain a deeper understanding of the regulatory mechanisms of LDO in macrophages at the genetic level, we performed RNA transcriptome sequencing of BMDMs. The analysis revealed significant differences in gene expression between the LPS-treated and LPS + LDO-treated groups (Fig. S4A). The strong correlation coefficients between samples within each group demonstrated the robustness and reproducibility of our data (Fig. S4B). Using GO and KEGG enrichment analyses, we found that the differentially expressed genes were primarily enriched in the cytokine–cytokine receptor interaction pathway following LDO treatment (Fig. 6A and B). Notably, inflammation-related cytokine and chemokine signaling pathways were significantly enriched, whereas glycolytic processes were down-regulated after LDO treatment (Fig. S4C). These results are consistent with our previous observations and provide further genetic evidence for the mechanistic role of LDO in modulating macrophage function.

Based on these findings, we hypothesized that LDO influences macrophage activity by regulating cytokine-associated pathways, ultimately affecting the pathogenesis and progression of sepsis. Furthermore, to delve deeper into the specific regulatory effects of LDO on macrophages, we analyzed gene expression within the cytokine–cytokine receptor interaction pathway (Fig. 6C). Notably, our results indicated that genes associated with chemokine signaling were significantly down-regulated. Consequently, chemokine-related signaling pathways, including chemokine-mediated signaling pathway, chemokine activity, cell chemotaxis, and CCR chemokine receptor binding pathways, were all down-regulated after LDO treatment (Fig. 6D to G and Fig. S4D), further corroborating its regulatory role in suppressing chemokine signaling.

To decipher the critical inflammatory mediators within the macrophage microenvironment during sepsis, further studies focused on receptor–ligand (R–L) interactions [37,38]. We conducted a literature review to confirm ligand expression in macrophages and identified R-L pairs based on expression correlations to map intercellular communication networks (Fig. 6H). This approach identified several critical pairs, including CXCRs-Cxcls, IFNRs-Ifns, and CCRs-Ccls, whose ligands were exclusively produced by macrophages. Therefore, these findings suggest that LDO regulates macrophage-mediated inflammatory responses by modulating chemokine-based R–L interactions.

Furthermore, we observed a marked up-regulation of M2 macrophage markers at the transcriptional level in the LDO-treated group, suggesting that LDO facilitates macrophage polarization toward the M2 phenotype under inflammatory conditions (Fig. S4E). In addition, to further investigate the upstream regulatory pathways governing these inflammatory mediators, we systematically evaluated the NLRP3 inflammasome signaling cascade. The NLRP3 inflammasome, composed of the adaptor apoptosis-associated speck-like protein (ASC) and NLRP3, is a critical regulator of pro-inflammatory cytokine secretion [39]. WB and IF experiments revealed that LDO treatment significantly inhibited NLRP3 inflammasome activation in macrophages (Fig. 6I and J). In addition, the ROS levels were significantly reduced (Fig. S4F). Similar results were observed in PBMCs, peritoneal macrophages (PMs), and tissues, indicating that LDO suppresses the activation of the NLRP3 inflammasome, thereby inhibiting systemic inflammatory storms in septic models (Fig. S4G and H).

The RNA-seq data confirmed that LDO promoted macrophage polarization toward the anti-inflammatory M2 phenotype, subsequently suppressing sepsis-induced excessive inflammation. By modulating cytokine signaling pathways, LDO mitigates the cytokine storm, ultimately reducing organ damage and improving sepsis treatment (Fig. 6K). This regulation mitigates the excessive inflammatory response associated with sepsis and offers a potential therapeutic mechanism.

### LDO-induced macrophage polarization: Shifting from pro-inflammatory M1 to anti-inflammatory M2 phenotypes

Having revealed that LDO holds significant potential for regulating macrophage polarization phenotypes, a series of detailed experiments was conducted thoroughly to assess whether macrophage polarization could be transformed from the M1 to the M2 phenotype.

**Fig. 7.**
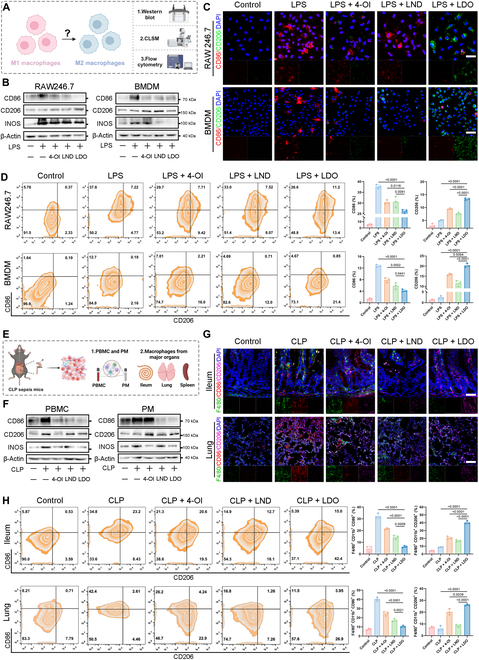
LDO-induced macrophage polarization: shifting from pro-inflammatory M1 to anti-inflammatory M2 phenotypes. (A) Experimental establishment for macrophage phenotype characterization in RAW264.7 and BMDM model. (B) Western blot for CD86, CD206, and INOS was performed after treatment of 4-OI, LND, or LDO in RAW264.7 cells and BMDMs. (C) Immunofluorescence of CD86 and CD206 in RAW264.7 cells and BMDMs. Scale bars: 30 and 50 μm. (D) CD86 and CD206 expression in RAW264.7 cells and BMDMs (*n* = 3). (E) Experimental establishment for macrophage phenotype characterization in PBMCs, PMs, ileum tissues, lung tissues, and spleen tissues. (F) Western blot for CD86, CD206, and INOS was performed in PBMCs and PMs. (G) Immunofluorescence of CD86 and CD206 in ileum and lung tissue. Ileum scale bars: 50 μm. Lung scale bars: 25 μm. (H) CD86 and CD206 expression in ileum and lung tissue (*n* = 3).

Initially, we conducted detailed experiments to investigate whether LDO can regulate macrophage polarization phenotypes in vitro (Fig. 7A). After LDO treatment, there was substantial up-regulation of M2 macrophage markers in RAW264.7 and BMDMs, including CD206, and a concomitant down-regulation of M1 markers, such as CD86 and inducible nitric oxide synthase (INOS) [11,40] (Fig. 7B). IF assays (Fig. 7C) and flow cytometry (Fig. 7D) precisely showed similar trends of CD86 and CD206 expression in RAW264.7 and BMDMs.

To comprehensively and systematically evaluate the in vivo impact of LDO on macrophage polarization, we established a CLP mouse model of sepsis (Fig. 7E)*.* PBMCs and PMs were firstly isolated to assess macrophage phenotypes in the circulatory system and the peritoneal cavity, revealing a significant reduction in M1 macrophages and an increase in M2 macrophages (Fig. 7F). These findings further substantiate the hypothesis that LDO induces a shift toward the anti-inflammatory M2 phenotype in macrophages, reducing cellular damage and mitigating cytokine storms within the circulatory system, ultimately alleviating sepsis-related tissue damage in the mouse model.

In addition, we collected major organs from the mice (ileum, lung, and spleen) and evaluated macrophage polarization phenotypes using IF (Fig. 7G) and flow cytometry (Fig. 7H and Fig. S5A). The significant reduction in F4/80 following LDO treatment indicated decreased overall inflammatory macrophage infiltration within the tissues. Moreover, the number of M2 macrophages increased significantly, whereas the proportion of M1 macrophages decreased markedly. Therefore, these observations indicate that LDO broadly activates the anti-inflammatory capabilities of macrophages in various organs, promoting the transition from M1 to M2 macrophages, thus ameliorating excessive organ damage associated with sepsis.

### LDO inhibits CCL2-driven cytokine storm in mice with sepsis

**Fig. 8.**
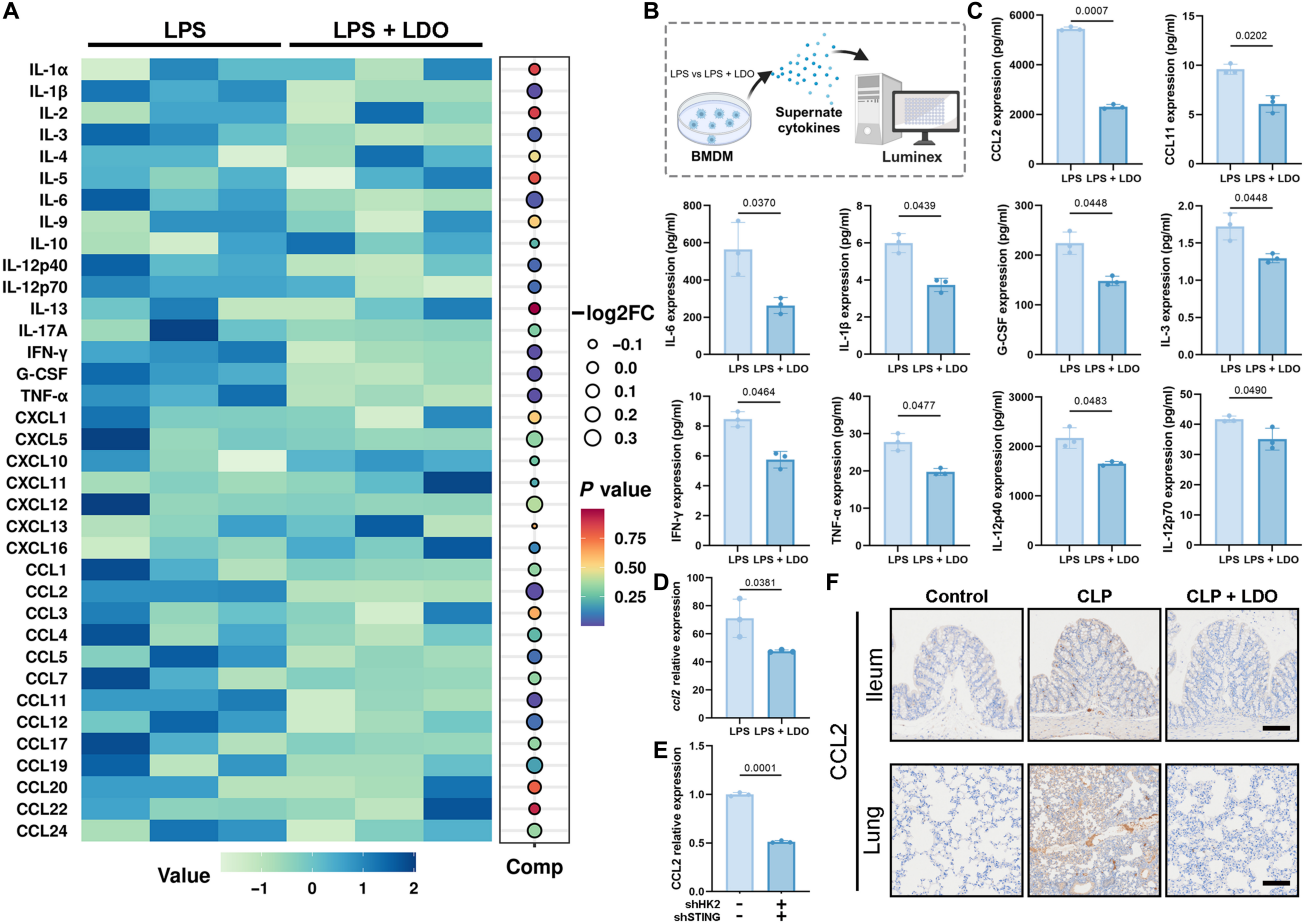
LDO inhibits CCL2-driven cytokine storm in sepsis. (A) Heatmap of BMDMs’ cytokines after treatment of LPS or LPS + LDO. (B) Luminex analysis for macrophage cytokines in BMDM supernatants. (C) The expression of 10 cytokines changed most significantly. (D) Relative expression of *ccl2* in the transcriptome level. (E) CCL2 expression of RAW 264.7 or co-knockdown RAW264.7 after LPS treatment. (F) Immunohistochemistry of CCL2 in ileum and lung tissue. Ileum scale bars: 40 μm. Lung scale bars: 80 μm.

To comprehensively assess the impact of LDO on macrophage-mediated inflammatory cytokine secretion, we conducted a comprehensive analysis of the cytokines in the supernatants of BMDMs. The Luminex results revealed a substantial cytokine reduction from the interleukin and chemokine families following LDO treatment (Fig. 8A and B). This reduction correlated with the decreased cytokine levels observed in vivo. Statistical analysis of the multiplex cytokine data revealed that CCL2 exhibited the most pronounced alteration among all the cytokines assessed (Fig. 8C).

We also examined the down-regulation of CCL2 at the transcriptional level (Fig. 8D). Furthermore, the co-knockdown of HK2 and STING resulted in a marked decrease in CCL2 levels (Fig. 8E), supporting the hypothesis that LDO mitigates the CCL2-driven inflammatory cytokine storm by inhibiting these pathways. However, a significant down-regulation of CCL2 in the ileum and lung tissues provided robust in vivo evidence for the suppressive effect of LDO on this cytokine (Fig. 8F). These findings collectively suggest that LDO inhibits the CCL2-driven cytokine storm, ultimately attenuating the excessive inflammatory response characteristic of sepsis.

### LDO alleviates sepsis and pulmonary injury by modulating CCL2-mediated macrophage infiltration and polarization

**Fig. 9.**
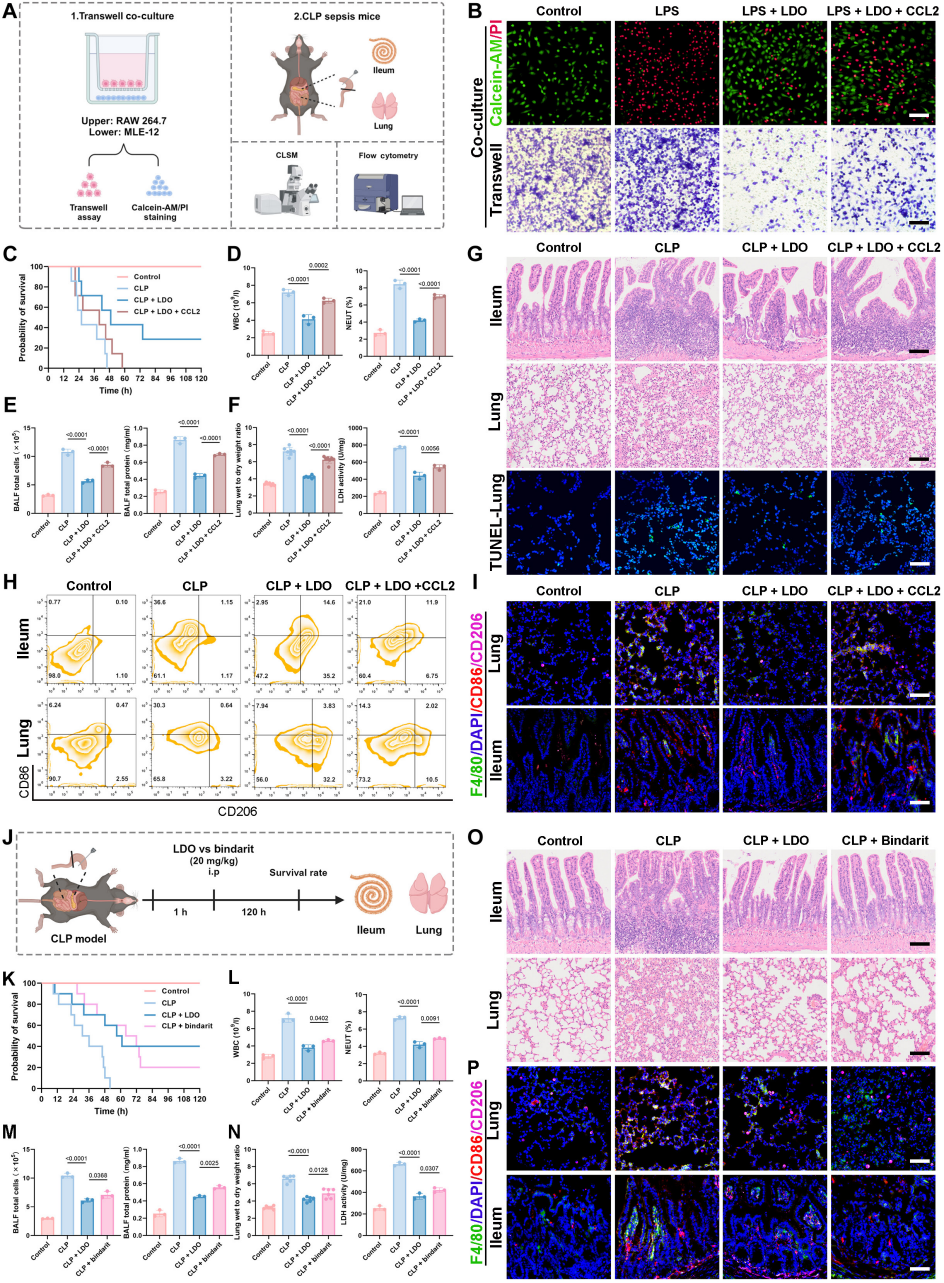
LDO alleviates sepsis and pulmonary injury by modulating CCL2-mediated macrophage infiltration and polarization. (A) Schematic overview of in vivo and in vitro experimental design. (B) Macrophage migration of RAW 264.7 cells and Calcein AM/PI staining assay of MLE-12 cells. Scale bars: 120 μm. (C to F) Survival rate (*n* = 7), blood routine (*n* = 3), BALF (*n* = 3), wet/dry ratio (*n* = 7), and LDH activity (*n* = 3) were measured. (G) Representative images of H&E staining of ileum and lung tissue, and TUNEL staining of ileum tissue. Scale bars: 120, 80, and 65 μm. (H) CD86 and CD206 expression in ileum and lung tissue (*n* = 3). (I) Immunofluorescence of CD86 and CD206 in ileum and lung tissue. Ileum scale bars: 50 μm. Lung scale bars: 25 μm. (J) Schematic overview of *in vivo* experimental design after treatment of LDO or Bindarit. (K to N) Survival rate (*n* = 10), blood routine (*n* = 3), BALF (*n* = 3), wet/dry ratio (*n* = 6), and LDH activity (*n* = 3) were measured. (O) Representative images of H&E staining of ileum and lung tissue. Scale bars: 120 and 80 μm. (P) Immunofluorescence of CD86 and CD206 in ileum and lung tissue. Ileum scale bars: 50 μm. Lung scale bars: 25 μm.

CCL2 is closely associated with immune activation and inflammation and is crucial in macrophage recruitment and activation at inflammatory sites, thereby amplifying the inflammatory response [41]. Therefore, we used in vitro and in vivo models to clarify how LDO mitigates sepsis and the associated pulmonary injury through a CCL2-dependent pathway (Fig. 9A).

Initially, a transwell co-culture system was used to evaluate the effect of LDO-treated macrophages on migration and anti-inflammatory potential (Fig. 9B). Addition of exogenous CCL2 enhanced RAW264.7 cell migration and increased MLE-12 cell death, confirming LDO’s regulation of macrophage behavior via a CCL2-dependent mechanism. In vivo, CCL2 counteracted the anti-inflammatory effects of LDO, worsened sepsis, and reduced survival (Fig. 9C). Blood and liver function tests confirmed that CCL2 exacerbated systemic inflammation and liver damage (Fig. 9D and Fig. S6A). Lung tissue analysis further revealed that CCL2 reversed the therapeutic effects of LDO by increasing inflammatory cell infiltration and triggering a cytokine storm, ultimately aggravating pulmonary edema (Fig. 9E and F) and oxidative damage (Fig. S6B). H&E and TUNEL staining showed a similar trend (Fig. 9G). Flow cytometry and IF results demonstrated that CCL2 increased M1 macrophage infiltration in ileum (Fig. 9H and I and Fig. S6C) and lung tissues (Fig. S6D), indicating that LDO regulates macrophage infiltration and phenotype through a CCL2-dependent mechanism.

In addition, to further assess CCL2 inhibition, Bindarit [42], a CCL2 inhibitor, was used as a positive control (Fig. 9J). Bindarit showed initial efficacy, whereas LDO surpassed it in reducing systemic inflammation (Fig. 9K and L), protecting liver function (Fig. S6E), and mitigating lung injury (Fig. 9M and N and Fig. S6F). H&E staining confirmed that LDO and Bindarit reduced damage to ileal and lung tissues (Fig. 9O). However, IF analysis revealed that LDO mainly promoted M2 macrophage polarization, whereas Bindarit primarily reduced M1 macrophages (Fig. 9P). These findings demonstrate that the superior efficacy of LDO over Bindarit is due to its ability to modulate macrophage polarization and suppress inflammatory cytokines, offering a strong mechanistic rationale for its therapeutic effects.

## Discussion

Immunotherapy is rapidly becoming a transformative approach in sepsis treatment [43–45]. Among the diverse immune cells within the septic inflammatory milieu, macrophages play a dominant role in driving the progression of sepsis and the dysregulation of immune homeostasis [46,47]. Our study underscores the pivotal roles of HK2 and STING in orchestrating the inflammatory response in sepsis and provides effective guidance for sepsis treatment.

Sepsis-induced macrophage polarization is marked by a striking shift toward the pro-inflammatory M1 phenotype, which induces coagulation dysfunction, inflammation, and immune disorders [48–51]. In this study, LDO disrupted HK2-mediated glycolytic flux while dampening STING-driven innate immunity signaling, ultimately orchestrating the profound reprogramming of macrophages from a destructive M1 state to a reparative M2 phenotype.

Itaconate, a natural metabolite, exhibits potent immunomodulatory effects by targeting the STING-mediated inflammatory pathways in macrophages [52]. However, LDO solves several shortcomings of itaconate, including its poor bioavailability, nonspecificity, and inhibition under hyperactive glycolysis. The ROS response design of LDO enables it to selectively target the inflammatory microenvironment. Therefore, by inhibiting macrophage glycolysis, LDO significantly enhances the anti-inflammatory efficacy of itaconate and optimizes the in vivo therapy.

Mechanistically, by robustly attenuating STING signaling cascades and HK2-driven glycolysis, LDO actively down-regulates macrophage chemokine signaling pathways, especially CCL2, thus opening new avenues for targeted interventions in sepsis management.

Moreover, unlike typical CCL2 inhibitors, our study takes a different approach by focusing on the metabolic reprogramming of macrophages to modulate CCL2 expression at its source. Notably, LDO demonstrated superior efficacy compared with the CCL2 inhibitor Bindarit in septic models. We postulate that the short-term efficacy of Bindarit (0 to 36 h post-CLP surgery) is due to its direct suppression of CCL2. However, the inability of Bindarit to sustain the long-term inhibition of macrophage CCL2 release or induce M2 polarization underscores the superiority of LDO.

In conclusion, this study elucidates the central roles of HK2 and STING in metabolic reprogramming during sepsis therapy. We developed and synthesized a novel nanoparticle, LDO, which showed powerful anti-inflammatory properties by disrupting HK2-mediated glycolytic flux and suppressing STING-induced type I interferon responses. This metabolic recalibration drives a pronounced shift in macrophage polarization from a pro-inflammatory M1 phenotype to a reparative M2 state, with down-regulations of CCL2-dependent cytokine storms and systemic inflammation. Therefore, our in vivo results validate the capacity of LDO to preserve organ integrity, and bioinformatics and clinical analyses highlight the potential applications extending to sepsis immunotherapy.

Notably, in the future, the development of LDO will open new avenues for designing innovative nanoparticle strategies aimed at manipulating macrophages to reshape the septic immune landscape.

## Methods

### Cell lines and cell culture

RAW 264.7 cells were obtained from Wuhan Pricella Biotechnology (China), grown in Dulbecco’s modified Eagle’s medium (DMEM) (Sangon, Shanghai, E600028). MLE-12 cells were purchased from Shanghai Fusheng Industrial (China), grown in DMEM/F12 (Servicebio, G4612). Two media were supplemented with 10% FBS (Excell Bio, Shanghai, FSP500) and 1% PS (MacGene, Beijing, CC040). All cell lines were maintained in a CO₂ incubator (Thermo Fisher, Forma 371) at 37 °C with 5% CO₂.

### BMDM extraction

BMDMs were isolated from 6-week-old female C57BL/6 mice and maintained in complete DMEM containing macrophage colony-stimulating factor (M-CSF) (20 ng/ml, Sino Biological) at 37 °C under 5% CO₂. Following a 7-day incubation period, adherent cells were identified as BMDMs and harvested for further experimental use.

### Animals

Healthy female C57BL/6 mice (6 to 8 weeks old, weighing 18 to 20 g) were acquired from Augct Bio, Xi’an. The animals were maintained in a pathogen-free environment, 6 per cage, under controlled conditions: temperature at 21 ± 2 °C, relative humidity between 40% and 70%, and a 12-h light/dark cycle.

### Clinical samples

Healthy controls were selected from individuals undergoing routine health checkups at the First Affiliated Hospital of Xi’an Jiaotong University. Sepsis patients were diagnosed based on the criteria outlined in The Third International Consensus Definitions for Sepsis and Septic Shock (Sepsis-3.0). The inclusion criteria for sepsis patients were as follows: (a) evidence of confirmed infection, (b) presence of secondary organ dysfunction or acute worsening of pre-existing organ dysfunction, and (c) a Sequential Organ Failure Assessment score of ≥2. Exclusion criteria included the following: (a) age below 18 years or above 65 years, (b) mortality within 24 h of intensive care unit admission, (c) diagnosis of autoimmune diseases or congenital immunodeficiency, and (d) presence of hematological disorders.

### Preparation and characterization of nano-assemblies

LDO was dissolved in dimethyl sulfoxide and introduced dropwise into deionized water under vigorous stirring. The colloidal stability of LDO was evaluated in PBS. The hydrodynamic diameter of LDO was measured using a Zetasizer (Nano ZS, Malvern Co., UK), while its morphology was examined by transmission electron microscopy (HITACHI, HT7700, Japan).

### Western blot

Cells were plated in 6-well plates and exposed to various drug solutions for 24 h. Protein extraction was performed using radioimmunoprecipitation assay buffer, followed by separation via sodium dodecyl sulfate–polyacrylamide gel electrophoresis and subsequent transfer to polyvinylidene difluoride membranes. The membranes were blocked with 5% nonfat milk in TBST (Tris-buffered saline with Tween 20) for 1 h at room temperature. After blocking, the membranes were incubated with primary antibodies overnight at 4 °C, washed 5 times with TBST, and then treated with secondary antibodies for 1 h at room temperature. Finally, the membranes were immersed in ECL substrate, and images were captured using a chemiluminescence imaging system. Details of all antibodies used are provided in Table S5.

### RNA-seq analysis

BMDMs were cultured in 60-mm dishes and exposed to LPS (1 μg/ml) either alone or together with LDO (50 μM) for 36 h. Total RNA extraction was carried out using TRIzol reagent (Thermo Fisher, 15596018). RNA quality was verified using the Bioanalyzer 2100 system and the RNA 6000 Nano LabChip Kit (Agilent, CA, USA, 5067-1511), and only samples with an RNA integrity number exceeding 7.0 were processed for library construction. RNA-seq was performed by LC Sciences on the Illumina X10 platform (Hangzhou, Zhejiang, China). Genes meeting the criteria of a false discovery rate < 0.05 and an absolute fold change ≥ 2 were classified as differentially expressed. These genes were further analyzed for functional enrichment through GO and KEGG pathway analysis.

### Flow cytometry

For macrophages (from lung, ileum, and spleen), the cells were isolated and enzymatically dissociated using collagenase IV and DNase I at 37 °C for 30 min. The resulting cell lysate was filtered through a 70-μm mesh to obtain a single-cell suspension. Cell counts were determined, and aliquots were prepared at the desired density for antibody staining. For cell lines, cells were washed and harvested prior to staining. All cells were incubated with blocking antibodies for 30 min, followed by staining with surface markers. For intracellular staining, cells were first fixed with 4% paraformaldehyde (PFA), permeabilized, and then incubated with antibodies diluted in permeabilization buffer after surface staining. Detailed information on all antibodies used is provided in Table S3.

### ELISA

The cell culture medium was centrifuged at 3,000 rpm for 10 min, and the supernatant was collected for analysis. Lung tissues were minced into small pieces and lysed on ice for 1 h. Serum samples were obtained by centrifuging coagulated blood twice at 5,000 rpm and 4 °C. All samples were processed according to the manufacturer’s protocols. The ELISA kits used in this study are listed in Table S4.

### Intracellular ROS and live and death cell measurements

LPS (1 μg/ml) was incubated with cells over 2 h before treated with LND (50 μM), 4-OI (50 μM), and LDO (50 μM) over 24 h. On the basis of standard ROS assay kit (Beyotime, Shanghai, S0033S) and Calcein-AM/PI apoptosis kit (Dojindo, C542) instructions, a probe was added (2 ml per well) and cells were incubated for another 15 min. The cells were exposed to the laser at 488 nm detected by a confocal laser scanning microscope.

### Immunofluorescence

Cells were plated on glass slides and treated with LPS (1 μg/ml) alone or in combination with drugs (4-OI, LND, LDO, and Bindarit, each at 50 μM) or LPS + LDO + CCL2 (500 nM). After treatment, cells were fixed with 4% PFA and blocked with a solution containing 1% bovine serum albumin and 22.52 mg/ml glycine in PBS with 0.1% Tween 20. Primary antibodies targeting CD86, CD206, NLRP3, p-STING, and HK2 were applied and incubated overnight at 4 °C, followed by 5 washes with TBST. Secondary antibodies were then incubated for 1 h at room temperature. Detailed information on all antibodies used is provided in Table S5.

### Multiplex IF staining

Tissue sample sections were fixed and blocked the same as IF. Antibodies targeting F4/80 were applied and incubated overnight at 4 °C. After 5 washes, primary antibodies targeting CD86 (Rat) and CD206 (Rabbit) were used. Secondary antibodies with Cy3 (Rat) and Alexa Fluor 647 (Rabbit) were then incubated for 1 h at room temperature. Detailed information on all antibodies used is provided in Table S5.

### Immunohistochemistry

Following a 1-h blocking step, the tissue (lung and ileum) sections were incubated with the CCL2 primary antibody at 4 °C overnight. Antigen localization was visualized using an avidin–biotin peroxidase detection system with 3,3'-diaminobenzidine (DAB) as the substrate. Images were captured using a conventional light microscope.

### Sepsis model

(a) CLP-induced sepsis model: C57BL/6 mice were anesthetized, shaved, and disinfected. A midline incision exposed the cecum, which was ligated at 30% and punctured with a 21-gauge needle. Feces were expelled to confirm patency. The cecum was returned, and the incision was closed with clips. Mice received subcutaneous saline for resuscitation. Sham mice had cecal manipulation without ligation/puncture. Tramadol (10 mg/kg, i.p.) was given twice daily. Mortality was monitored for 5 days. (b) LPS-induced sepsis model: C57BL/6 mice were intravenously injected with LPS (15 mg/kg) and observed for 5 days.

### Treatment of sepsis mice

C57BL/6 mice were randomly divided into control (PBS), LPS/CLP, and LPS/CLP + drugs (4-OI, LND, and LDO) groups. C57BL/6 mice were intraperitoneally injected with PBS, 4-OI (20 mg/kg), LND (20 mg/kg), LDO (20 mg/kg), Bindarit (20 mg/kg), and CCL2 (5 mg/kg) after LPS/CLP challenge. All the drugs were dissolved in PBS. The serum levels of cytokine were measured 12 h after LPS/CLP challenge. The tissues (ileum and lung) were assembled for H&E staining.

### Statistical analysis

Data are presented as mean ± SD. Group comparisons were assessed using 2-tailed unpaired *t* tests, while differences among multiple groups were evaluated by one-way analysis of variance followed by Tukey’s post hoc test. All statistical analyses were conducted using GraphPad Prism software (version 9). A *P* value < 0.05 was considered statistically significant, with significance levels denoted as *P < 0.05, **P < 0.01, and ***P < 0.001.

### Ethical Approval

Ethics approval was granted by the Ethics Committee of The First Affiliated Hospital of Xi’an Jiao Tong University (XJTU1AF2024LSYY-366) and Animal Experiment Center of Northwestern Polytechnical University Animal Care and Use Guidelines (202301197). Raw data for the RNA-seq of LDO-treated BMDM cells and bulk RNA-seq of BMDM cells have been deposited in GEO (GSE279691).

## Data Availability

All data are available in the main text or the Supplementary Materials.

## Supplementary Materials

Supplementary 1 Scheme S1 Tables S1 to S5 Figs. S1 to S6
