# Supplementary material for: A Self-Assembled Metabolic Regulator Reprograms Macrophages to Combat Cytokine Storm and Boost Sepsis Immunotherapy
Source: Research (Wash D C). 2025 Apr 1;8:0663. doi: 10.34133/research.0663 (PMC11959697; doi:10.34133/research.0663)
Supplement: Supplementary 1 — Scheme S1 Tables S1 to S5 Figs. S1 to S6 [file research.0663.f1.pdf]

# Supplementary Material

## A Self-Assembled Metabolic Regulator Reprograms Macrophages to Combat Cytokine Storm and Boost Sepsis Immunotherapy

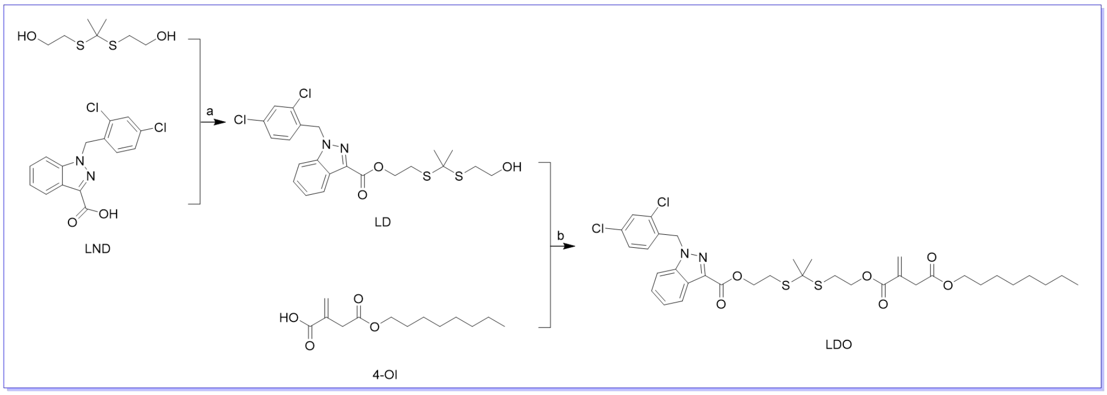


**Scheme S1. Synthesis of prodrug LDO.** Reagents and conditions: (a) EDCI, DMAP, DMF, rt, 12h; (b) EDCI, DMAP, DMF, rt, 12h.

*Chemical materials:* Lonidamine (LND), 1-Ethyl-3-(3-dimethylaminopropyl) carbodiimide (EDCI), 4-Dimethylaminopyridine (DMAP), 2,2'-(Propane-2,2-diylbis (sulfanediyl))diethanol, 4-Octyl itaconate (4-OI), Bindarit were purchased from Aladdin Biochemical Technology Co. Ltd. (Shanghai, China). $^1$H and $^{13}$C NMR spectra were recorded on a Bruker ARX 500 MHz spectrometer. High-resolutionmassspectra (HRMS) were obtained on a Thermo Scientific Q ExactivePlus LC/MS mass spectrometer.

*Synthesis for LD:* 2,2'-(Propane-2,2-diylbis(sulfanediyl))diethanol (0.12 mmol) was dissolved in 4 ml of dimethyl sulfoxide (DMSO) in a 50-ml round-bottom flask. Then, LND (0.12 mmol) and DMAP (0.48 mmol) were added dropwise sequentially and stirred in an ice bath for 30 minutes. Next, EDCI (0.36 mmol) was added, and the mixture was further stirred at room temperature for 24 hours. The completion of the reaction was monitored by thin-layer chromatography. The target products were

purified by column chromatography to give a Transparent oil like ***LD*** (76 mg, Yield, 61%). $^{1}H$ NMR (500 MHz, $CDCl_3$): δ 8.27 (d, *J* = 8.1 Hz, 1H), 7.47 – 7.40 (m, 2H), 7.39 – 7.32 (m, 2H), 7.09 (dd, *J* = 8.4, 2.1 Hz, 1H), 6.70 (d, *J* = 8.3 Hz, 1H), 5.79 (s, 2H), 4.64 (t, *J* = 7.2 Hz, 2H), 3.79 (t, *J* = 6.1 Hz, 2H), 3.12 (t, *J* = 7.2 Hz, 2H), 2.88 (t, *J* = 6.1 Hz, 2H), 1.67 (s, 6H), 1.25 (s, 1H). $^{13}C$ NMR (126 MHz, $CDCl_3$) δ 162.23, 140.82, 135.60, 134.57, 133.03, 132.04, 129.46, 129.41, 127.69, 127.54, 123.92, 123.66, 122.51, 109.76, 63.87, 61.44, 56.36, 50.43, 33.81, 31.19, 29.17.

*Synthesis for LDO:* 4-Octyl itaconate (4-OI) (0.24 mmol) was dissolved in 4 ml of dimethyl sulfoxide (DMSO) in a 50-ml round-bottom flask. Then, LD (0.24 mmol) and DMAP (0.48 mmol) were added dropwise sequentially and stirred in an ice bath for 30 minutes. Next, EDCI (0.36 mmol) was added, and the mixture as further stirred at room temperature for 24 hours. The completion of the reaction was monitored by thin-layer chromatography. The target products were purified to give a Transparent oil like substance ***LDO*** (133 mg, Yield, 71%). $^{1}H$ NMR (500 MHz, $CDCl_3$) δ 8.25 (dd, *J* = 8.1, 1.1 Hz, 1H), 7.43 – 7.31 (m, 4H), 7.07 (dd, *J* = 8.4, 2.1 Hz, 1H), 6.69 (d, *J* = 8.4 Hz, 1H), 6.32 (s, 1H), 5.77 (s, 2H), 4.61 (t, *J* = 7.2 Hz, 2H), 4.31 (t, *J* = 7.0 Hz, 2H), 4.07 (t, *J* = 6.8 Hz, 2H), 3.31 (s, 2H), 3.09 (t, *J* = 7.2 Hz, 2H), 2.91 (t, *J* = 7.0 Hz, 2H), 1.65 (s, 8H), 1.32 – 1.23 (m, 10H), 0.86 (t, *J* = 6.8 Hz, 3H).$^{13}C$ NMR (126 MHz, $CDCl_3$) δ 170.67, 165.86, 162.16, 140.81, 135.63, 134.54, 133.73, 133.02, 132.08, 129.43, 128.69, 127.67, 127.48, 123.88, 123.62, 122.51, 109.74, 65.16, 64.02, 56.63, 50.42, 37.70, 31.77, 31.07, 29.20, 29.18, 29.16, 29.03, 28.55, 25.85, 22.64, 14.10.

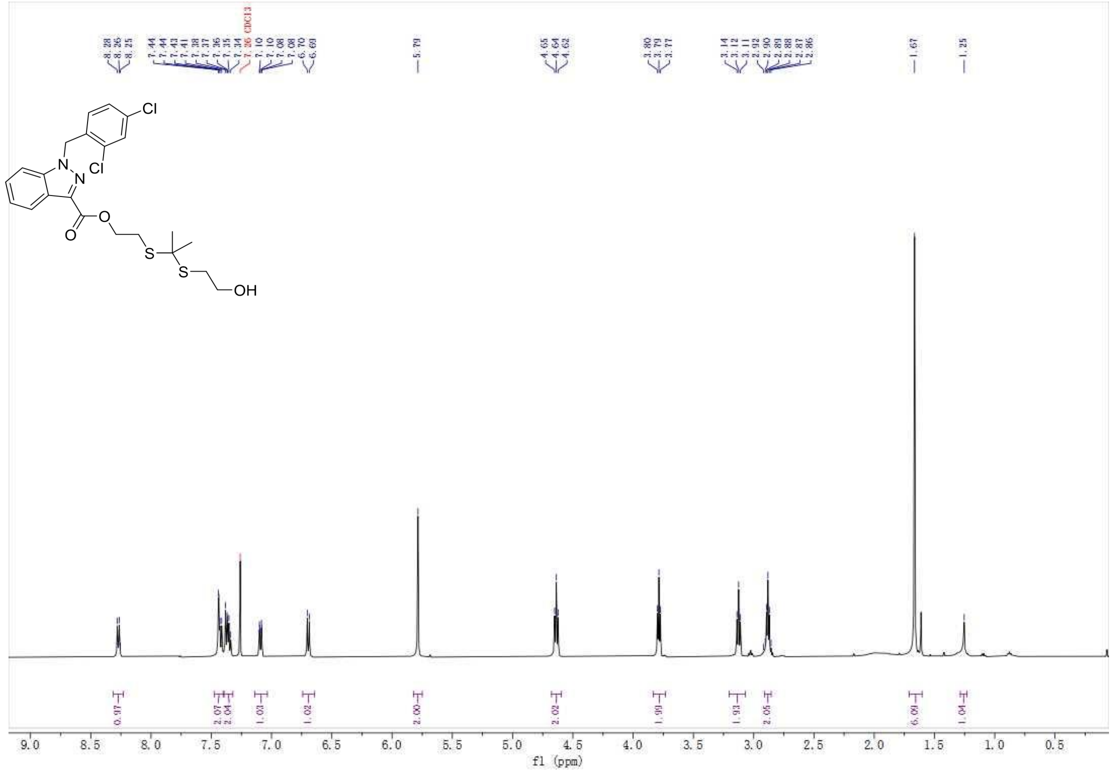

$^{1}$H-NMR specture of LD

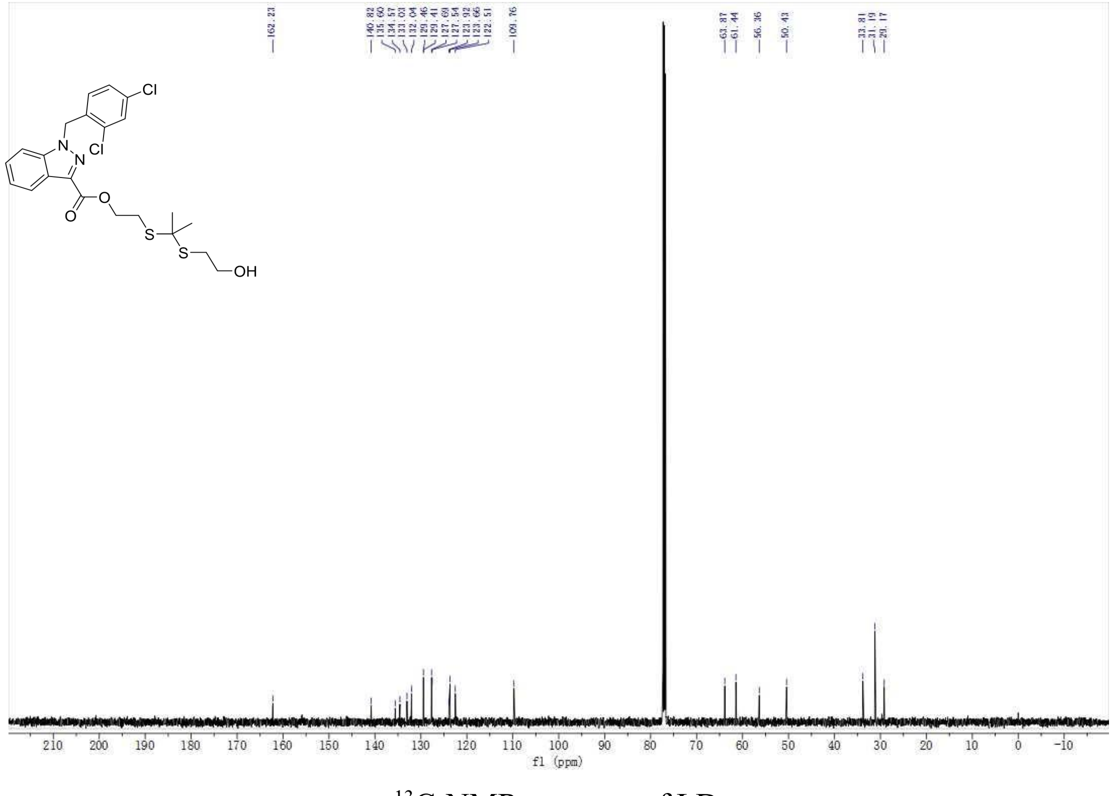

$^{13}$C-NMR specture of LD

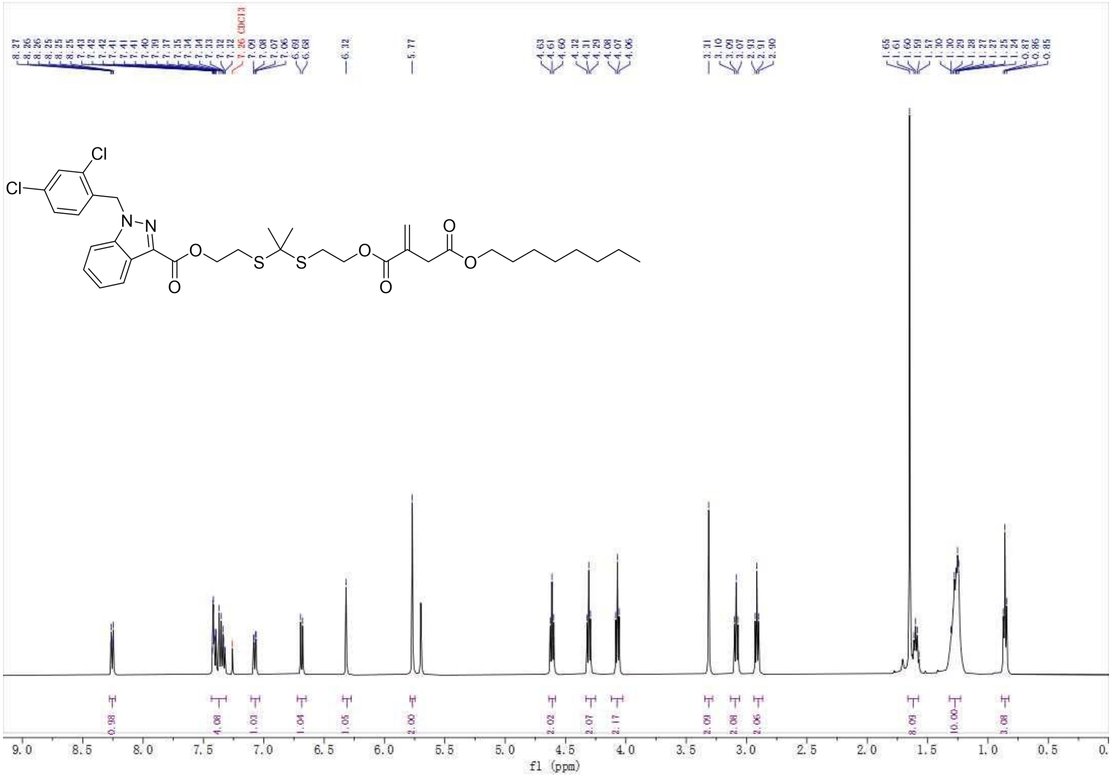

$^{1}$H-NMR specture of LDO

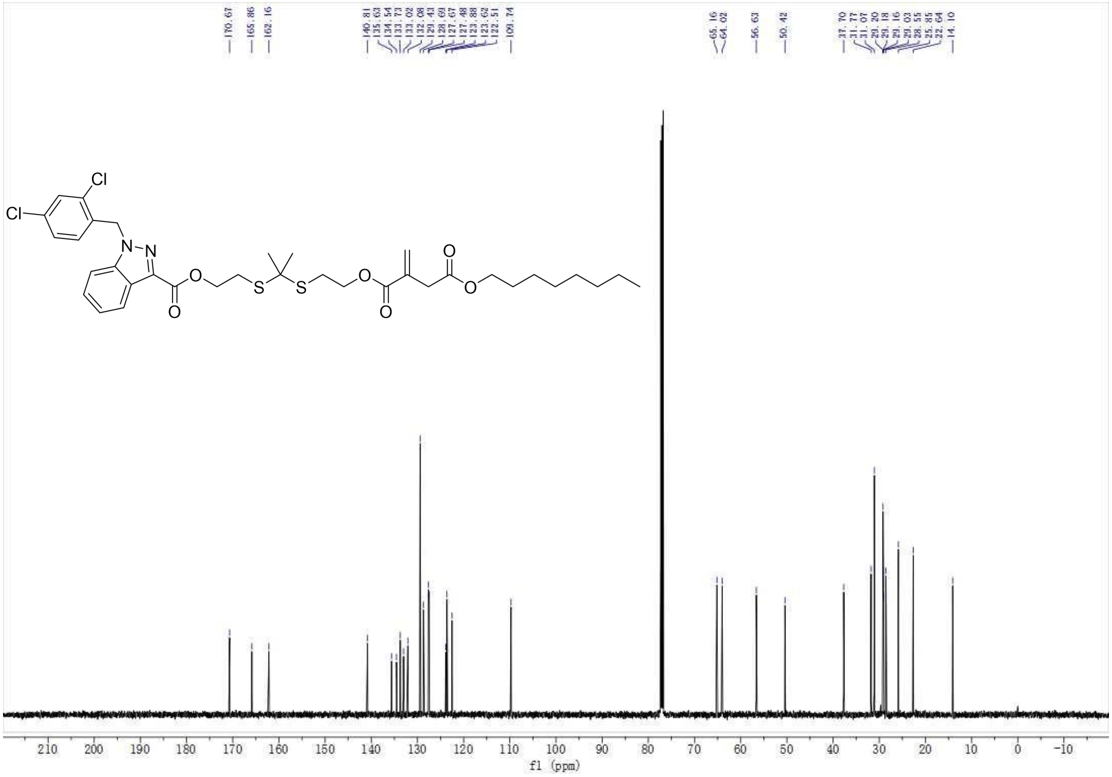

$^{13}$C-NMR specture of LDO

**Supplementary Table S1. shRNA sequences targeting STING.**

| Target | Sequence |
|---|---|
| Mouse shSTING#1 | CCGGATGATTCTACTATCGTCTTATCTCGAGATAA GACGATAGTAGAATCATTTTTT |
| Mouse shSTING#2 | CCGGCAACATTCGATTCCGAGATATCTCGAGATA TCTCGGAATCGAATGTTGTTTTT |
| Mouse shSTING#3 | CCGGAGAGGTCACCGCTCCAAATATCTCGAGATA TTTGGAGCGGTGACCTCTTTTTT |

**Supplementary Table S2. shRNA sequences targeting HK2.**

| Target | Sequence |
|---|---|
| Mouse shHK2#1 | CGGTACAGAGAAAGGAGACTT |
| Mouse shHK2#2 | GCCAACTTCATGGACAAGCTA |
| Mouse shHK2#3 | GCATATGATCGCCTGCTTATT |
| Mouse shHK2#4 | GCAGTAATGCCTGCTACATGG |

**Supplementary Table S3. Antibodies used for flow cytometry.**

| Antibody | Dilution | Catalog | Manufacturer |
|---|---|---|---|
| APC Anti-Mouse CD86 (GL1) | 1:100 | Apc-65068 | Proteintech |
| CD11b/ITGAM (M1/70) Rat mAb (PerCP-Cy5.5® Conjugate) | 1:80 | 85601 | CST |
| FITC anti-mouse F4/80 Antibody | 1:200 | 123108 | Biolegend |
| PE anti-mouse CD206 (MMR) Antibody | 1:40 | 141706 | Biolegend |

**Supplementary Table S4. ELISA assay kits.**

| Antibody | Catalog | Manufacturer |
|---|---|---|
| The Mouse IFN-γ ELISA Kit | 88-7314-88 | Thermo Fisher |
| The Mouse IFN-β ELISA Kit | 424001 | Thermo Fisher |
| The Mouse IL-6 ELISA Kit | 88-7064-88 | Thermo Fisher |
| The Mouse TNF-α ELISA Kit | 88-7324-88 | Thermo Fisher |
| The Mouse CCL2 ELISA Kit | 88-7391-22 | Thermo Fisher |
| The Mouse IL-10 ELISA Kit | 88-7105-22 | Thermo Fisher |
| The Mouse IL-1β ELISA Kit | 88-7013-22 | Thermo Fisher |

**Supplementary Table S5. Antibodies used for IF, IHC and Western blot.**

| Antibody | Catalog | Manufacturer |
|---|---|---|
| Anti-Vinculin antibody | ab129002 | Abcam |
| beta Actin Rabbit antibody | ab8226 | Abcam |
| STING (D1V5L) Rabbit mAb | 50494 | CST |
| Phospho-STING (Ser365) (D8F4W) Rabbit mAb | 72971 | CST |
| Phospho-STING (Ser365) (D1C4T) Rabbit mAb | 51865 | CST |
| cGAS (D3O8O) Rabbit mAb | 31659 | CST |
| TBK1/NAK (D1B4) Rabbit mAb | 3504 | CST |
| Phospho-TBK1/NAK (Ser172) (D52C2) Rabbit mAb | 5483 | CST |
| IRF-3 (D83B9) Rabbit mAb | 4302 | CST |
| Phospho-IRF-3 (Ser396) (D6O1M) Rabbit mAb | 29047 | CST |
| NLRP3 Rabbit antibody | ab270449 | Abcam |
| ASC/TMS1 Rabbit Antibody | 67824 | CST |
| HK2 Rabbit antibody | ab209847 | Abcam |
| CD86 Rabbit antibody | 97778 | CST |
| CD86 Rat antibody | ab238468 | Abcam |
| CD206/MRC1 (E6T5J) XP® Rabbit mAb | 24595 | CST |
| iNOS Rabbit antibody | ab178945 | Abcam |
| F4/80 (D4C8V) XP® Rabbit mAb (Alexa Fluor® 488 ) | 27076 | CST |
| Anti-MCP1 antibody | ab315478 | Abcam |
| Anti-rabbit IgG, HRP-linked Antibody | 7074 | CST |
| Goat Anti-mouse IgG H&L | ab6708 | Abcam |
| Goat Anti-Rabbit IgG H&L | ab205718 | Abcam |
| Goat Anti-Rabbit IgG H&L (Alexa Fluor® 647) | ab150079 | Abcam |
| Goat Anti-Mouse IgG H&L (Alexa Fluor® 647) | ab150115 | Abcam |
| Goat Anti-Rabbit IgG H&L (Alexa Fluor® 488) | ab150077 | Abcam |
| Goat Anti-Rabbit IgG H&L (Cy3®) | ab97075 | Abcam |
| Goat Anti-Rat IgG H&L (Cy3®) | GB21302 | Servicebio |

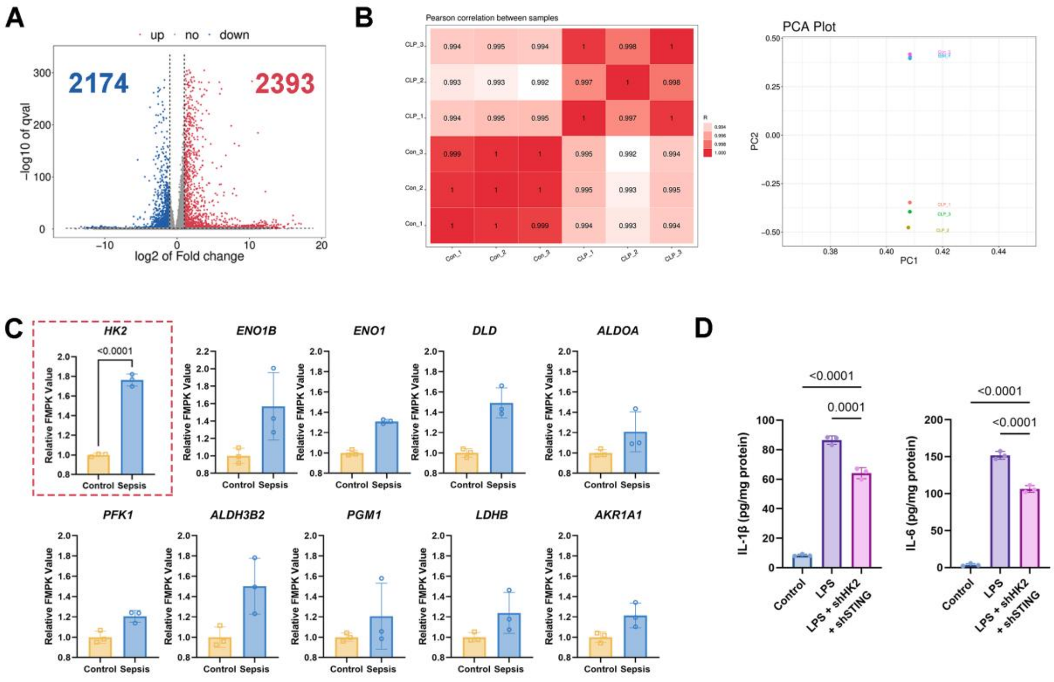


**Figrue S1**. (A) Volcano plot of differentially expressed genes. (B) Pearson correlation between samples and their PCA plot. (C) Expressions of key genes involved in glycolytic process. (D) The level of IL-1β and IL-6 secretion (n = 3).

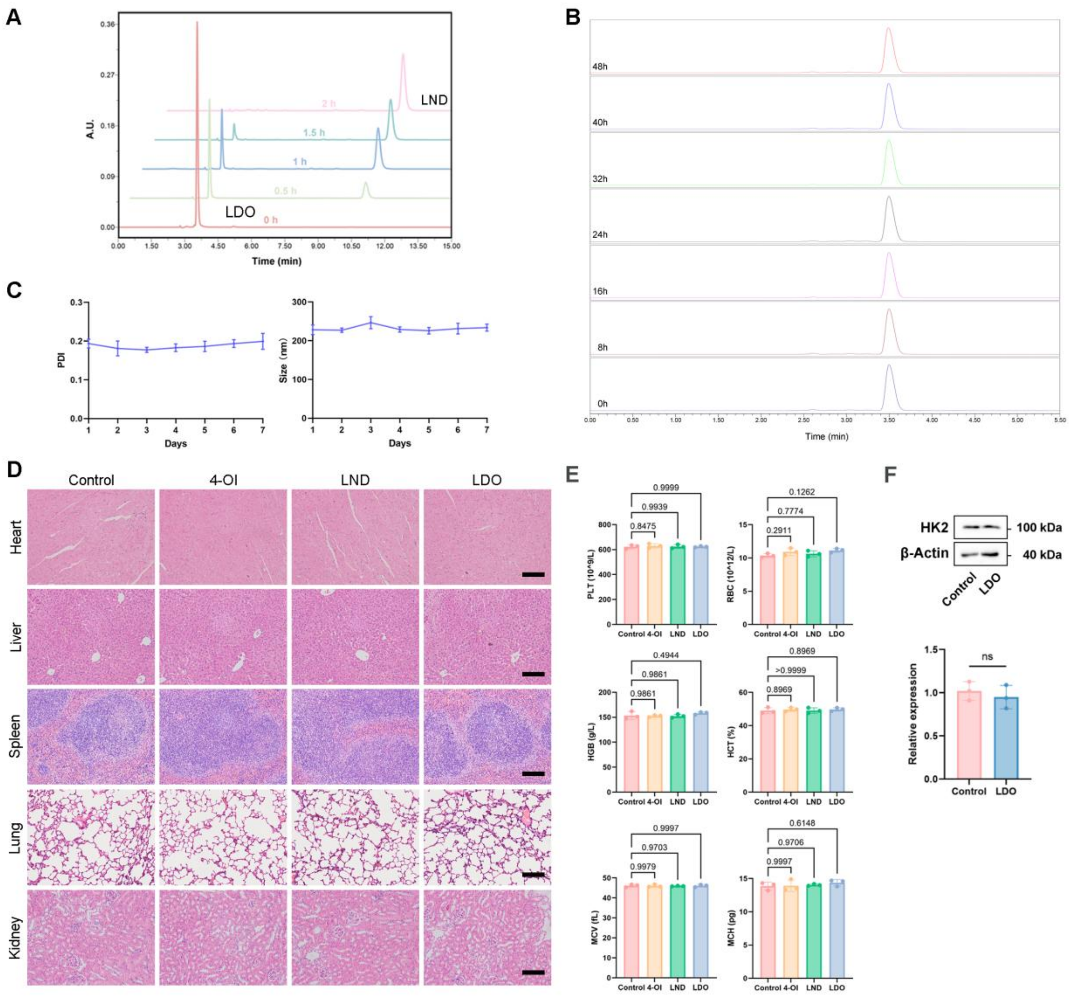


**Figure S2.** (A) The degradation of LDO tested by HPLC. System: 95% ACN + 5% $H_2O$. (B) The stability of LDO tested by HPLC. System: 95% ACN + 5% $H_2O$. (C) Stability analysis of nanoparticles by measuring average sizes and PDI of nanoparticles in aqueous environments by DLS for 7 days (n = 3). (D) HE staining of vital organs (heart, liver, spleen, lung, kidney). Scale bars: 100 µm. (E) Several blood index values (n = 3) for *in vivo* safety of compounds. (F) Western blot for HK2 expression in lung tissues was performed after LDO treatment.

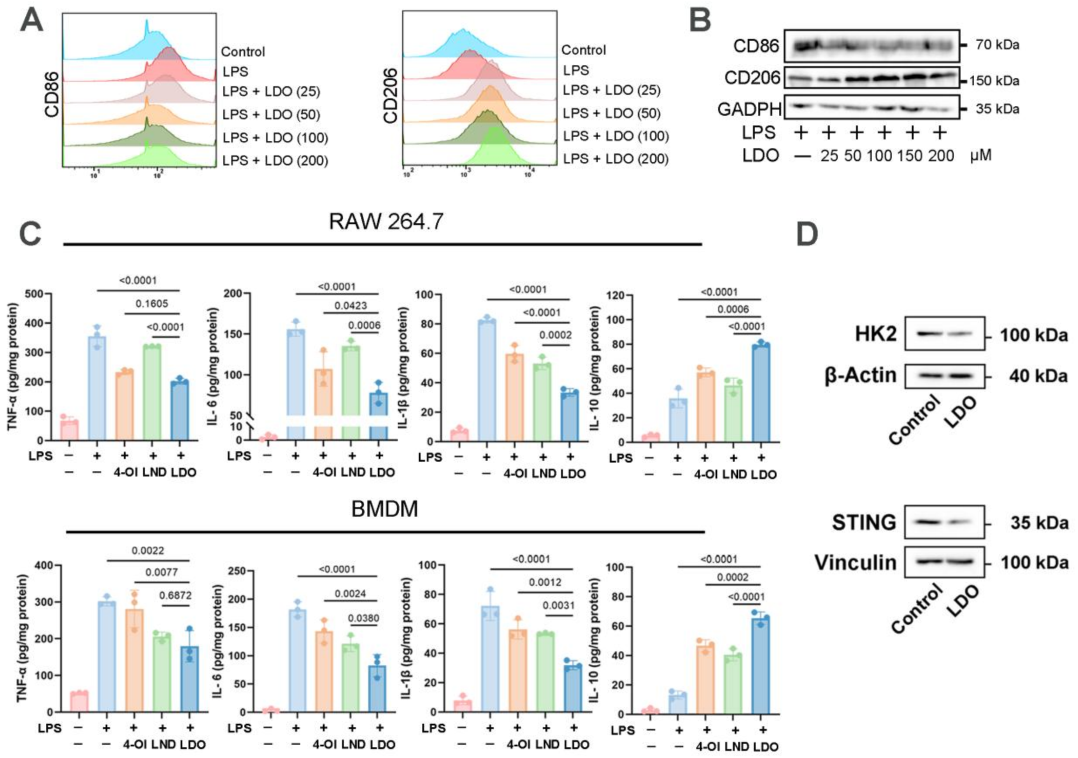


**Figrue S3**. (A) CD86 and CD206 expression in RAW264.7 after different dose of LDO tested by flow cytometry. (B) Western blot of CD86 and CD206 in RAW264.7 after different dose of LDO. (C) ELISA was used to measure TNF-α, IL-6, IL-1β，IL-10 in medium (n = 3). (D) Western blot for HK2 and STING was performed with or without LDO in PBMCs in sepsis mice.

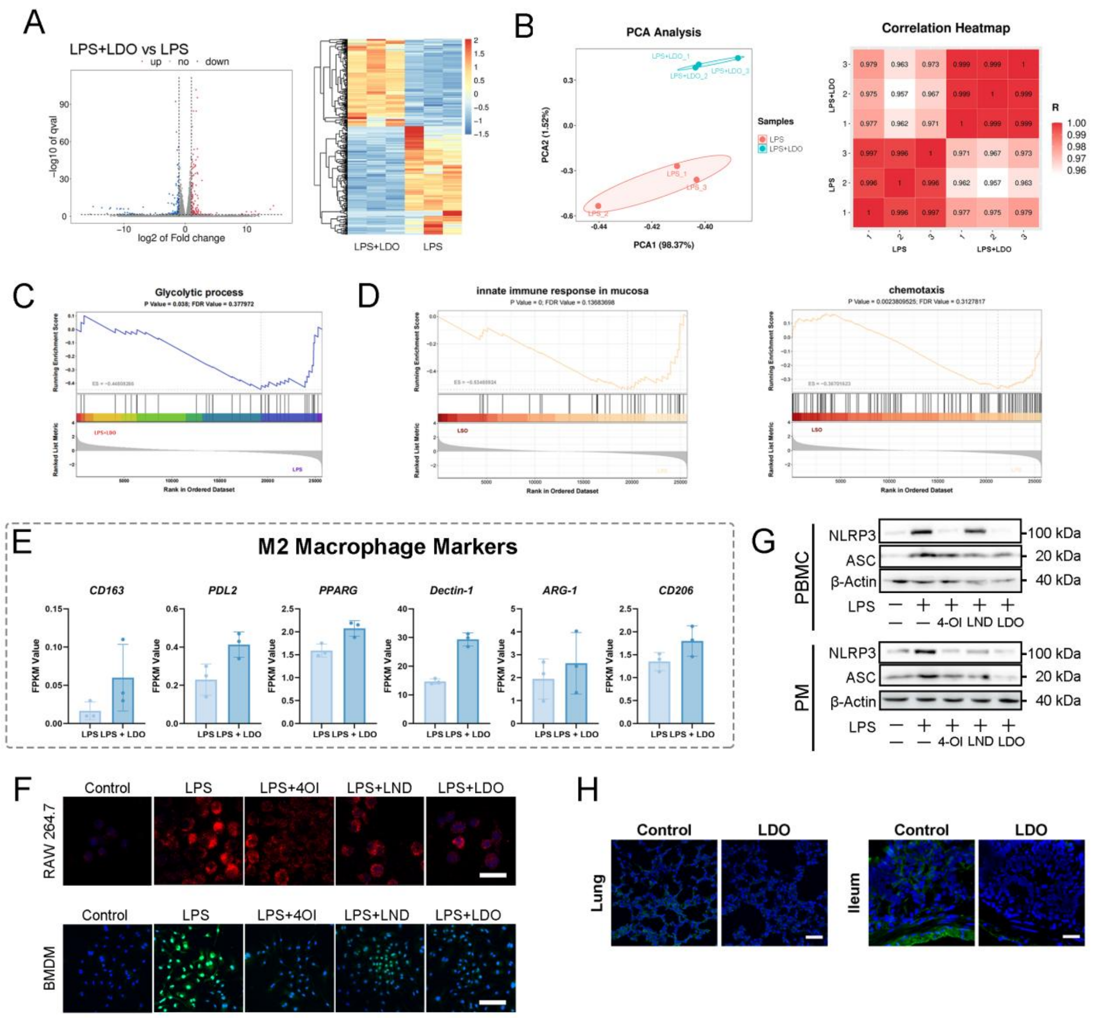


**Figrue S4**. (A) Volcano plot of differentially expressed genes. Heatmap of differentially expressed genes after LDO + LPS treatment or LPS treatment (n = 3). (B) Pearson correlation between samples and their PCA plot. (C) GSEA analysis of genes in glycolytic process. (D) GSEA analysis of genes in chemotaxis and innate immune response in mucosa. (E) Expressions of M2 macrophage markers. (F) Measuring cellular ROS by fluorescence microscopy. RAW264.7: scale bars: 30 μm. BMDM: Scale bars: 50 μm. (G) Western blot for NLRP3 and ASC in PBMC and PM. (H) Immunofluorescence of NLRP3 in ileum and lung tissue. Ileum scale bars: 50 μm. Lung scale bars: 25 μm.

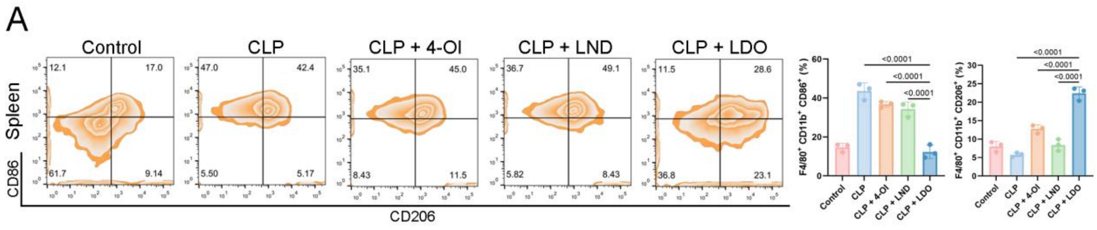


**Figure S5**. (A) CD86 and CD206 expression in spleen tissue (n = 3).

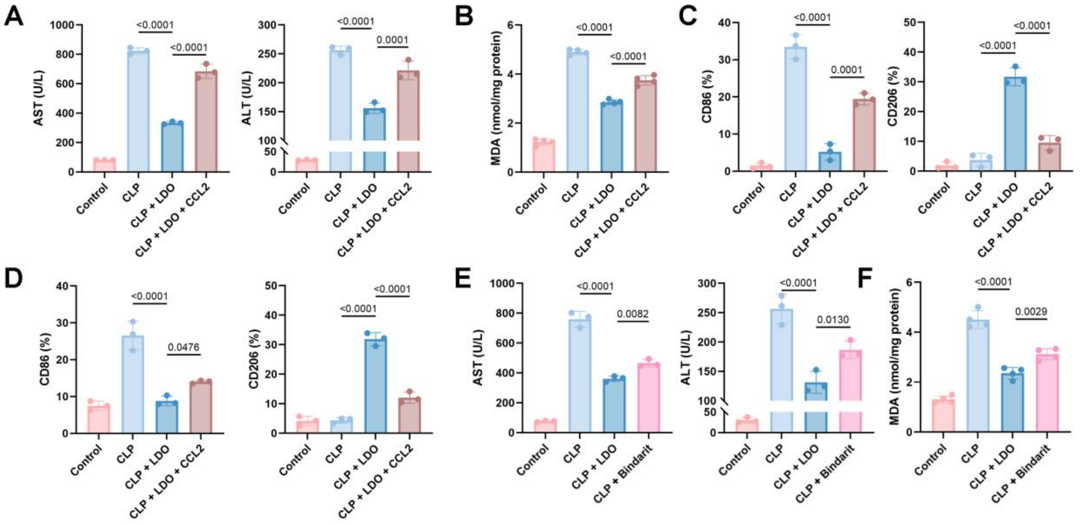


**Figrue S6**. (A) Liver function examinations (n = 3) were measured after treatment with PBS, LDO and LDO + CCL2. (B) MDA level of lung tissues were measured (n = 4). (C) Expression of CD86 and CD206 in ileum tissues (n = 3). (D) Expression of CD86 and CD206 in lung tissues (n = 3). (E) Liver function examinations (n = 3) were measured after treatment with PBS, LDO and LDO + Bindarit. (F) MDA level of lung tissues were measured (n = 4).
